# Implementation of External Beam Five-Fraction Adjuvant Breast Irradiation in a US Center

**DOI:** 10.3390/cancers14061556

**Published:** 2022-03-18

**Authors:** Jacob Eckstein, Peter Taylor, Ruqin Zheng, Lucille Lee, William Chen, Louis Potters, Clary Evans

**Affiliations:** 1 Department of Radiation Medicine, Northwell Health Cancer Institute, Lake Success, NY 11042, USA; jeckstein3@northwell.edu (J.E.); ptaylor3@northwell.edu (P.T.); rzheng3@northwell.edu (R.Z.); llee3@northwell.edu (L.L.); wchen8@northwell.edu (W.C.); lpotters@northwell.edu (L.P.); 2 Zucker School of Medicine, Hofstra University, Hempstead, NY 11549, USA

**Keywords:** breast radiation, ultrahypofractionation, hypofractionation

## Abstract

**Simple Summary:**

Radiation therapy to the whole breast is standard of care following breast-conserving surgery for early breast cancer. Previously this treatment was delivered daily over 3–5 weeks. Clinical trials of shorter treatment protocols completing radiation in five treatments have been completed showing similar outcomes to longer protocols; however, there remains a lack of international consensus as to which patients are best treated with shorter regimens. Here we describe how we developed a consensus in our department for using a five-fraction regimen, using a survey of physicians and a review of the most relevant literature.

**Abstract:**

Five-fraction adjuvant whole breast radiation has been shown to be a safe and effective alternative to longer fractionation regimens. Given the lack of international consensus on patient selection for the protocol, we developed a consensus protocol to guide patient selection and facilitate safe and efficient five-fraction radiation in our radiation medicine department. In developing the directive, we surveyed departmental physicians about their choice of adjuvant breast regimen for various clinical scenarios. Patient travel burden was the factor most strongly impacting radiation oncologists’ decision-making when considering prescribing a five-fraction course of adjuvant breast radiation; the length of clinical trial follow-up data and acute and late normal tissue effects also impacted it, along with personal clinical experience and experience of dosimetry and physics personnel. Relative value unit (RVU) reimbursement and financial toxicity to the patient were reported to be less important in decision-making. Physicians were most comfortable using five-fraction radiation in women >50 years of age with low-risk cancer and for patients unable to attend for longer treatment courses. Eight months after implementation, the protocol accounts for 4.7% of breast irradiation delivered in our department.

## 1. Introduction

Adjuvant radiation following breast conserving surgery halves the breast cancer recurrence rate and reduces the breast cancer death rate by about one-sixth [1]. Consequently, adjuvant radiation to the whole breast following breast conserving surgery is the standard of care in the United States (US) and globally. Previously, this was delivered as a 5-week course of daily radiation; this “standard” fractionation delivered 23–28 fractions of radiation at 1.8–2 Gy per fraction. Moderate hypofractionation was shown to be non-inferior to standard fractionation [2,3,4,5,6] and adjuvant radiation to the whole breast is now more often delivered in 15 or 16 fractions of 2.6–2.7 Gy, as per the American Society of Radiation Oncology (ASTRO) guidelines [7]. European Society for Radiation and Oncology Advisory Committee for Radiation Oncology Practice (ESTRO-ACROP) guidelines now support the use of five-fraction whole breast radiation as a standard of care; the guidelines recommend that nodal irradiation be given using moderate hypofractionation [8]. 

As a result, many centers in Europe have adopted 26 Gy in five fractions as a standard of care for all indications of whole-breast, partial-breast and chest-wall radiation [9,10]. A lack of international consensus on patient selection for the regimen has limited its uptake in the US, but such a consensus is now emerging. For example, five-fraction adjuvant radiation is listed in the 2022 National Comprehensive Cancer Network (NCCN) guidelines version 2.2022 as an alternative for select patients over 50 years of age with pTis/T1/T2/N0 breast cancer. Therefore, many US centers are now offering five-fraction radiation to select patients [11]. Whilst ultrahypofractionation may not be suitable for all patients [12], it offers a safe and effective alternative to longer protocols, with fewer treatment visits, reduced overall machine time, fewer travel requirements, less need for patients to request time away from work or caring responsibilities and offers reduced costs to healthcare providers and patients compared with longer regimens.

The move towards ultrahypofractionation is subsequent to the publication of two randomized controlled trials conducted the United Kingdom (UK) [13,14,15,16] and a phase two non-randomized trial in the US [17,18]. Key trial design features of the randomized controlled trials are detailed in Table 1.

Our academic radiation medicine department is distributed over nine treatment locations in New York serving a highly diverse community with significant healthcare disparities. It was determined that offering ultrahypofractionated radiation may offer significant benefit to some patients with breast cancer, especially those for whom no radiation is the only alternative due to medical or social barriers to care [19] and those looking to accelerate and shorten their care. The implementation was expedited, because fewer hospital visits also offered a tangible advantage during the COVID-19 pandemic [20]. 

The majority of patients treated in our radiation medicine department are treated in accordance with the standards set by a library of default departmental treatment directives. Departmental treatment directives serve as comprehensive documents detailing prescription dose, simulation instructions, target volume descriptions, dose-volume histogram objectives and priorities, special physics consult requests and on-treatment and nursing instructions, along with supporting references [21,22]. Physicians choose the most appropriate directive for each patient, and can modify the directive selectively as required for the needs of individual patients. The directive is integrated into the electronic medical record (EMR) of each patient which is peer-reviewed at chart rounds [23] along with the accompanying contours or treatment plan. In order for five-fraction whole breast radiation to be delivered to patients in the department, a new treatment directive was developed, as was the case with implementation of moderate hypofractionation [24]. 

## 2. Materials and Methods

First, in order to survey opinion of departmental physicians to build a consensus for the directive, a fully anonymized electronic survey (Survey Monkey, San Mateo, CA, USA) was conducted of the radiation oncology physician faculty and residents in our department, assessing the current practice and attitudes towards five-fraction radiation. The survey questions were jointly developed by departmental radiation oncology attending physicians who treat breast cancer regularly. Invitations to participate in the survey were sent to 30 recipients, including resident and attending physicians. Respondents were asked how often they treat breast cancer (5-point answer scale from “Always” to “Never”) and whether they had previously prescribed a course of adjuvant whole breast radiation in five-fractions (Answer “Yes” or “No”). They were then asked “How often would you prescribe a five-fraction schedule for a patient: (1). >70 years old with low risk breast cancer? (2). 50–70 years old with low risk breast cancer? (3). <50 years old with low risk breast cancer? (4). With N1 disease? (5). Requiring post mastectomy radiation? (6). Requiring a more limited number of treatment visits?”. Answers were graded on a 5-point scale from “Always” to “Never.” Additionally, respondents were asked: “When considering a prescribing a five-fraction course of adjuvant breast radiation, the following factors impact my decision:” (1). Personal experience with five-fractions and organs at risk (OAR) constraints; (2). Dosimetry/physics experience with five-fractions and OAR constraints; (3). Length of long-term follow-up of clinical trials investigating five-fraction radiation; (4). Acute radiation-induced toxicity profile; (5). Chronic radiation-induced toxicity profile; (6). Ability of patient to attend treatment; (7). Financial toxicity to the patient; (8). Relative Value Unit (RVU) reimbursement. Answers were graded using a 5-point scale, from “strongly agree” to “strongly disagree”. 

Second, we surveyed the most relevant literature, in order to identify peer reviewed clinical trials studying five-fraction adjuvant whole breast radiation and summarize their results with respect to safety and efficacy, to inform patient selection criteria for the directive. Trials studying partial breast radiation and techniques other than external beam radiation were not included. To supplement this review, we included literature assessing the advantages and disadvantages of hypofractionation from an economic and healthcare equity perspective. There was no formal voting process but a consensus was reached through a series of meetings.

Using the results of the survey and the literature review, a draft directive was written following the Delphi technique in principle. Patient selection criteria for five-fraction radiation were detailed, reflecting both national guidelines and phase 3 clinical trial data, to be used to guide departmental decision-making. Radiation oncologists and representatives from dosimetry, medical physics and radiation therapists contributed to writing and approving the directive, which was then reviewed by members of the health system breast tumor board with relevant stakeholders including radiation oncologists, medical oncologists, breast surgeons and oncoplastic surgeons. 

Finally, we audited departmental utilization of five-fraction adjuvant radiation for breast cancer 8 months after the implementation of the treatment directive. All patients who received adjuvant whole breast radiation using any regimen from March 2021–October 2021 were reviewed. Patient counts were derived from the records of simulations over the time period and from a review of prescribed radiation doses. Patients who received regional nodal irradiation, post-mastectomy radiation therapy or partial breast irradiation or re-irradiation were not included in the analysis.

## 3. Results

### 3.1. Survey

A total of 15 out of 30 physicians responded to the survey:—13 attending physicians and 2 residents—with over 70% of respondents indicating that they treat breast cancer regularly. A total of 8 out of 15 respondents had previously prescribed a course of adjuvant whole breast radiation in five fractions.

**Figure 1:**
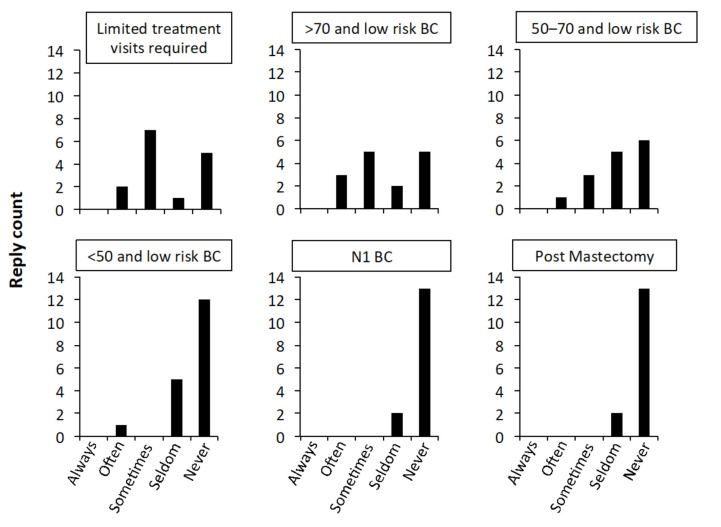
Responses from radiation oncologists describing how often they would prescribe a course of five-fraction radiation for six clinical scenarios. Numbers in panel titles indicate age ranges in years. BC–breast cancer; N1–axillary node positive.

When asked about the indication for five-fraction adjuvant RT for breast cancer, over half of the respondents indicated that they would offer this regimen either “often” or “sometimes” for patients who require a more limited number of treatment visits and for patients over the age of 70 years with low-risk breast cancer. In patients between 50–70 years with low risk breast cancer, around a quarter of the respondents indicated that they would give five-fraction breast radiation “often” or “sometimes.” The majority of the respondents indicated they would prescribe five-fraction radiation “seldom” or “never” in patients under 50 with low risk breast cancer, and in the node-positive or post-mastectomy setting; see Figure 1.

**Figure 2:**
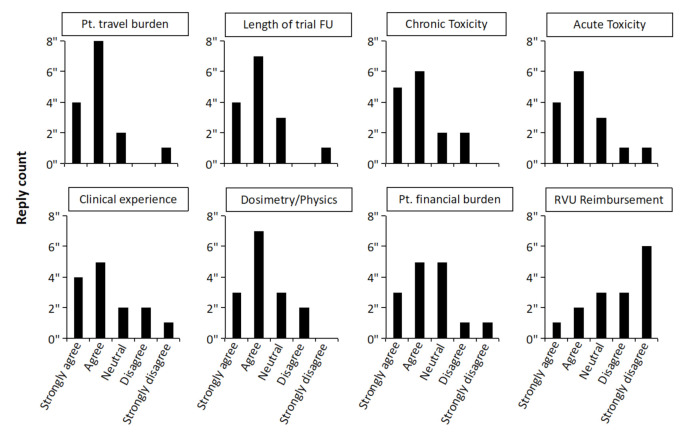
Responses from radiation oncologists describing factors that impact the decision to prescribe five-fraction adjuvant whole breast radiation therapy. Pt–patient; FU–follow up; RVU-–relative value unit.

Respondents then assessed eight factors for their impact on their decision to prescribe five-fraction radiation; see Figure 2. Taking “Strongly agree” and “Agree” together, the ability of patients to attend treatment was the factor most strongly impacting radiation oncologists’ decision-making when considering prescribing a five-fraction course of adjuvant breast radiation; the length of clinical trial follow-up data and acute and late normal tissue effects were also important, along with personal clinical experience and experience of dosimetry and physics personnel. Financial toxicity to the patient and RVU reimbursement were reported to be less important in decision-making. 

### 3.2. Patient Selection for the Five-Fraction Directive

Through the integration of the relevant literature, 2022 NCCN guidelines and the results of the departmental survey and peer feedback, we finalized our patient selection criteria for use in our department; see Table 2. 

Suitable patients for treatment with five-fraction adjuvant whole breast radiation are patients ≥ 50 years of age with lower risk (T1/T2 N0) breast cancer or ductal carcinoma in situ (DCIS) resected with margins ≥ 2 mm. Our departmental directive recommends caution in use of the five-fraction directive in the following settings: post-mastectomy, axillary node positive, T3-4, <50 years old or patients requiring a boost and axillary dissection > 8 nodes. Patients should be counseled on the very small risk of a less optimal cosmetic result, pending long-term follow-up from the FAST-Forward trial. The regimen may also be offered to select patients who may not meet the “suitable” criteria but who are unable to attend for longer dose/fractionation schemes due to medical or social constraints. The reasons for limited treatment visits being required might include poor performance status, co-morbidities, transportation difficulties, lack of social support, concern for lack of compliance with treatment visits and financial or insurance issues.

Whilst prospective studies validating five-fraction adjuvant breast RT did include boost options, the wide variety of boost fractionations in the literature and lack of widespread consensus on which boost schedule should follow a five-fraction protocol motivated the panel to include a boost requirement as a criterion for cautionary classification, especially since most patients requiring a boost are those at higher risk of recurrence, who will continue to be treated with moderate hypofractionation.

In our department, a boost is suggested for invasive disease with the following criteria: age < 50 with any grade disease or age 51–70 with high grade disease or positive margins or other high risk features including hormone receptor negativity or lymphovascular invasion. A boost may be omitted for patients meeting the following criteria: age > 70 with hormone receptor positive tumors of low to intermediate grade, resected widely with negative margins of ≥2 mm or patients aged 50–70 with extremely low risk disease. The boost prescription is 10 Gy delivered in four fractions using electrons or photons, depending on patient anatomy and the location of the tumor bed. Dose constraints for planning target volume (PTV) and OARs for the five-fraction directive were taken from the FAST-Forward protocol, published in the Supplementary Material of the trial [13].

### 3.3. Utilization

A total of 404 patients were treated with adjuvant whole breast radiation in the 8-month period after the five-fraction directive was implemented (March 2021–October 2021). In all, 382 (94.6%) patients were treated with moderate hypofractionation (16 fractions); 19 patients (4.7%) were treated with ultrahypofractionation (5 fractions) and 3 (0.7%) patients were treated with conventional fractionation (25 fractions). 

## 4. Discussion

This article details our experience of developing and implementing a five-fraction adjuvant breast radiation protocol in an academic multisite radiation medicine department in the US. There continue to be differing practices between individual physicians regarding the use of five-fraction radiation for breast cancer [11], as was also the case in our department. Following a departmental survey and literature review, a consensus, evidenced-based treatment directive was agreed upon. To standardize our practice to increase safety and efficiency, we built consensus around writing a directive for use across our sites as a default approach which would unify the fractionation scheme, set expectations for treatment planning and make it easy for community location physicians to incorporate this directive into their standard offering.

After the implementation of the directive, patients over 50 years of age with lower-risk (T1/T2 N0) breast cancer, or DCIS, can be offered five-fraction radiation. The consensus departmental default treatment option for patients with a higher risk of recurrence or higher risk of adverse events remains moderate hypofractionation, until 10-year follow-up data from FAST-Forward are available. We acknowledge that some patients with higher risk characteristics were recruited into FAST-Forward, albeit in small numbers, and patients requiring a fewer number of treatment visits may be offered five-fraction radiation despite not meeting the “suitable” criteria. There was hesitancy in offering the protocol to all patients, since the FAST trial was not powered for local control and long-term follow-up data for efficacy and normal tissue effects for 26 Gy in five daily fractions will only be available when the 10-year results from FAST-Forward trial are published.

Despite the development of a departmental directive, many treating physicians demonstrated hesitation to utilize ultrahypofractionated whole-breast radiation therapy, which led to a utilization rate of only 4.7% among patients receiving adjuvant whole breast radiation therapy over the first 8 months of the directive being in use. We anticipate increased adoption with increased time and familiarity with the directive. The recently updated NCCN guidelines, which now incorporate five-fraction radiation as a treatment option, and the expected longer follow-up data from the FAST-Forward clinical trial, are likely to increase confidence in the regimen. Additionally, five-fraction radiation is just one alternative among many for adjuvant radiation, including partial breast irradiation or the omission of radiation for the lowest-risk patients.

Participants in our survey did not consider value-based payment an important criterion when deciding to offer five-fraction adjuvant radiation therapy. Nevertheless, we recognize that the current reimbursement model means that five-fraction treatment impacts revenue and that this may present a barrier for implementation, especially in a fee-for-service payment system. The radiation oncology (RO) model announced by the Centers for Medicare and Medicaid Services is an Advanced Alternative Payment Model (APM) and a Merit-Based Incentive Payment System APM for modality-agnostic, episode-based payments. While implementation of the RO-APM is currently paused, implementation of changes to clinical practice, including the use of five-fraction radiation, may be facilitated by the absence of financial penalties for the health care provider. The five-fraction regimen is not designed as a one-size-fits-all approach to treating women with breast cancer; rather, it serves as one regimen among several, each of which outline specific eligibility criteria, albeit with some overlap, but with distinct indications.

The ability of patients to attend for treatment is important, as the respondents of our survey indicated, because the lack of adjuvant radiation is associated with decreased overall survival from breast cancer [19]. Impaired access to adjuvant radiation treatment has been shown to be more significant for elderly, rural, minority and uninsured patients in the US, especially those living further away from a treatment facility [25,26]. Medicare and Medicaid insurance, patients with an age of less than 50 years and patients from minority races have been shown to be at a higher risk of not achieving timely radiation completion for breast cancer, in an urban setting [27]. Reducing financial toxicity is a key intervention to reduce cancer health disparities [28] and offering hypofractionation is a key facility-directed recommendation to reduce potential financial toxicity [29]. Appropriately, overcoming barriers to radiation oncology access in low resource settings in the US has therefore been identified as a priority for equitable cancer care by the American Society of Radiation Oncology [30]. 

We acknowledge that our directive was developed by members of a single institution without a fully formalized Delphi process. The study is not intended to act as a consensus statement or guideline but rather a record of a single institution’s practice in the time before international consensus is reached. Particularly, the number of participants in the survey was small, which we acknowledge is a weakness of our study. We would note that this is inevitable when surveying a single institution, and that more half of the attending physicians in the department responded to the survey. Additionally, we acknowledge that the survey included both resident and attending physicians, when only attending physicians make final decisions for their patients. However, more than 85% of respondents were attending physicians, and residents also write the radiation prescriptions for patients, which are countersigned by the attending physician. We did not have access to a specialist librarian for the literature review and did not conduct a formal voting process to reach our consensus. Nevertheless, our directive matches the protocol of the FAST-Forward trial, a trial that established level-one treatment evidence, and is aligned with the NCCN and ESTRO-ACROP guidelines that were subsequently published. We have succeeded in the relatively early adoption of breast ultrahypofractionation in a US center, which offers advantages to select patients. 

## 5. Conclusions

In summary, informed by a departmental survey on its use, we implemented a five-fraction radiation protocol for patients with breast cancer in our department to facilitate its appropriate, efficient and safe use across our health system, for the benefit of our patients. We anticipate increased use of the directive in our center with time and hope that our work will aid other US radiation centers who are seeking to implement this treatment option for select patients.

## Figures and Tables

**Table 1:** Trial Design for FAST and FAST-Forward Trial [13,14,15,16,17].

	FAST	FAST-Forward
Timeframe	2004–2007	2011–2014
Sample Size	915	4096
Arms	50 Gy/2 Gy/5 weeks	40 Gy/2.67 Gy/3 weeks
	30 Gy/6 Gy/5 weeks	27 Gy/5.4 Gy/1 week
	28.5 Gy/5.7 Gy/5 weeks	26 Gy/5.2 Gy/1 week
Primary endpoint	Photographic appearance	Ipsilateral tumor recurrence
Statistical design	Non-inferiority	Non-inferiority
Eligibility Criteria	pT1–2 (<3 cm) pN0	pT1–3 pN0–1
	Age ≥ 50 years	Age ≥ 18 years
	BCS	BCS or mastectomy
	No chemotherapy	Chemotherapy allowed
Median follow-up	9.9 years	5.9 years

BCS: breast conserving surgery.

**Table 2:** Characteristics of Suitable Patients for 5-Fraction Whole Breast Irradiation.

Limited treatment visits required for medical or social reasons ^1^	OR	T1-2N0/DCIS ^2^ AND ≥50 years old AND Accepting potentially higher risk of toxicity beyond 5 years
		Cautionary characteristics: T3/T4 Node positive <50 years old Post mastectomy Boost required Axillary dissection > 8 nodes

^1^ Reasons for limited treatment visits being required might include: poor performance status, co-morbidities, transportation difficulties, lack of social support, concern for lack of compliance with treatment visits, financial or insurance issues. ^2^ DCIS: ductal carcinoma in situ.

## Data Availability

Data available upon request from the corresponding author.
